# Effects of oral nutritional supplements plus mature silkworm pill on cognitive function in Korean adults with mild cognitive impairment: a 12-week randomized blinded clinical trial

**DOI:** 10.3389/fnut.2025.1605525

**Published:** 2025-10-28

**Authors:** Min Ji Kim, Sun Min Lee, Kyunghwa Sun, Yeon Hee Kim, Ye Jin Seo, Sun Ah Park, Hyoung Su Park, Hye-Jin Kim, Yoo Kyoung Park, So Young Moon

**Affiliations:** ^1^Department of Medical Nutrition, Graduate School of East-West Medical Nutrition, Kyung Hee University, Yongin, Republic of Korea; ^2^Department of Neurology, Ajou University School of Medicine, Suwon, Republic of Korea; ^3^Laboratory for Neurodegenerative Dementia, Department of Anatomy, Ajou University School of Medicine, Suwon, Republic of Korea; ^4^Neuroscience Graduate Program, Department of Biomedical Sciences, Ajou University Graduate School of Medicine, Suwon, Republic of Korea; ^5^R&D Division, Maeil Health Nutrition Co., Ltd., Pyeongtaek, Republic of Korea

**Keywords:** mild cognitive impairment, silkworm, nutritional supplements, memory, delayed

## Abstract

**Objective:**

This study aimed to determine whether mature silkworm pills plus commercial oral nutritional supplements (ONS) could improve cognitive function, especially in individuals with mild cognitive impairment (MCI).

**Methods:**

A total of 62 participants (70.6 ± 8.5 years, 48.4% female), aged ≥50 years diagnosed with mild cognitive impairment but not dementia, were randomly assigned into the 12-week ONS plus silkworm pill group (30 participants, commercial ONS with mature silkworm pills) or the placebo group (32 participants, placebo ONS containing protein, carbohydrates, and fats, with placebo pills containing dextrin). During the 12-week intervention, participants consumed the allocated pills and ONS once daily. Additionally, nutritional education related to the Mediterranean-DASH Intervention for Neurodegenerative Delay (MIND) diet therapy and proper eating habits was provided at weeks 0, 3, 6, and 9. The primary outcome was interval change in the Korean version of the Repeatable Battery for the Assessment of Neuropsychological Status (K-RBANS) after the 12-week intervention. Secondary outcomes included interval changes in nutrient intake, nutrition-related blood test results, nutritional quotient, and MIND score evaluation.

**Results:**

After 12 weeks, the interval change in the delayed memory domain score of the K-RBANS was improved in the ONS plus silkworm pill group (65.9 ± 19.0 vs. 70.5 ± 19.0, *p* = 0.011), and there was also a difference between the intervention and control groups (*p* = 0.018). The interval changes in the total scale and other cognitive domain scores of the K-RBANS were not statistically significant. There were no significant differences in the pre- and post-comparison results between the placebo and ONS plus silkworm pill groups in terms of other secondary outcomes, including nutrition-related blood test results, nutritional quotient, and MIND score.

**Conclusion:**

After the 12-week intervention, the ONS and silkworm pill group showed a significant improvement in delayed memory compared with the placebo group. However, no significant differences were observed in other cognitive domains or secondary outcomes, such as nutrient intake, blood test results, nutrition quotient, or MIND score adherence. These findings suggest that, while the combination may have a positive impact on specific cognitive functions, further research is needed to fully understand its potential benefits and mechanisms of action.

**Clinical Trial Registration:**

https://trialsearch.who.int/Trial2.aspx?TrialID=KCT0007304.

## 1 Introduction

A multi-domain lifestyle modification intervention, including cognitive intervention, physical exercise, vascular risk factor management, and nutritional intervention, represented by the Finnish Geriatric Intervention Study to Prevent Cognitive Impairment and Disability (FINGER) study, has already been proven effective in preventing and delaying dementia progression (1). Globally, multi-domain intervention programs to prevent dementia have been developed to suit the circumstances and characteristics of each country, and a network called Worldwide FINGERs (WW FINGERs) has been launched (2). The SoUth Korean study to PrEvent cognitive impaiRment and protect BRAIN (SUPERBRAIN) health through lifestyle intervention in at-risk older people, which was developed and completed in 2018, is a customized dementia prevention program registered on WW FINGERs on behalf of Korea, and its effectiveness has already been verified and applied in actual clinical settings (3, 4). These multi-domain non-pharmacological intervention programs are particularly important for mild cognitive impairment (MCI) and normal cognition, and the significance of nutritional intervention as a key component has been previously highlighted (5).

The SUPERBRAIN study developed a nutritional intervention program consisting of the Mediterranean-DASH Intervention for Neurodegenerative Delay (MIND) diet, which is effective in preventing dementia, and conducted nutritional education for 24 weeks. Sub-analysis suggested that nutrition may be involved in the pathophysiology of dementia and that patients with cognitive decline may have specific nutritional requirements (6). Because most patients with MCI already have dementia pathology in progress, there has been constant discussion that nutritional support may help improve cognitive function in patients with MCI. In this context, there has been a steady demand for the development of medical foods that enhance cognitive function. Numerous studies have investigated the potential of commercial oral nutritional supplements (ONS) and their impact on improving cognitive function (7–10).

Silkworms (*Bombyx mori*), particularly in their mature stage prior to metamorphosis, have been increasingly investigated for their potential neuroprotective and health-promoting effects. The powders of steamed and lyophilized mature silkworms have been shown to contain high levels of bioactive nutrients such as essential amino acids, unsaturated fatty acids, vitamins, minerals, polyphenols, and γ-aminobutyric acid (GABA), which are known to contribute to neurological function and cognitive health (11–13).

Moreover, recent *in vivo* studies using rodent models have demonstrated that supplementation with mature silkworm powder, also known as Hongjam (a.k.a. golden silkworm-based), improved memory and learning abilities. For example, silkworm-derived intervention enhanced performance in the Morris water maze and passive avoidance tests, showing effects comparable to or exceeding those of donepezil, a commonly prescribed cholinesterase inhibitor for dementia (12, 14). These results suggest that silkworm-based formulations may exert cognitive benefits by modulating neurotransmitter systems and reducing oxidative stress (15–17).

This study aimed to investigate the potential benefits of a nutritional intervention program involving golden silkworm pills plus commercial ONS.

## 2 Materials and methods

### 2.1 Participants

Individuals aged ≥50 years with MCI were recruited to participate in this 12-week nutritional intervention program, which ran from May 2022 to June 2023. The inclusion criterion was being at the MCI stage but not having dementia, with at least one of the following conditions: (1) no significant functional impairment in daily activities [Korean Instrumental Activities of Daily Living (K-IADL) score < 0.4] (18), (2) literacy, and (3) written consent to participate in the study. The exclusion criteria included a confirmed psychiatric disorder (e.g., major depressive disorder); dementia; other neurodegenerative diseases (e.g., Parkinson's disease); severe or unstable symptomatic cardiovascular disease; stent insertion or angioplasty within the past year; a diagnosis of incurable malignancy within the past 5 years; any other evidence of an unstable physical condition (e.g., acute or severe asthma, active gastric ulcer, severe liver disease, or kidney disease requiring hemodialysis); a history of food or medication allergies; any other medical conditions that may affect the clinical trial completion; illiteracy; severe hearing, visual loss, or communication difficulty that precludes the intervention and validation evaluation; taking two or more prescribed medications aimed at improving cognitive function (e.g., cholinesterase inhibitor and N-methyl-D-aspartate receptor antagonist); starting a new prescribed medication for cognitive enhancement or adjusting the dosage within the past 3 months; starting other health supplements within the past 3 months; and enrollment in other interventional or pharmacological studies. Patient taking only one cognitive enhancer were asked to maintain his/her medication.

### 2.2 Composition of the ONS and pills

The products (ONS, the golden-silkworm pills) used in this study were provided by Maeil Health Nutrition Co. Ltd. (Gyeonggi-do, Republic of Korea) and were used throughout the research process. Both the experimental and control products provided 150 kcal, consisting of 15 g of carbohydrates, 6 g of fat, and 9 g of protein and the remaining vitamin and mineral is stated in the Supplementary Table 1. All ONS formulations, including the placebo, underwent batch testing for nutrient composition and microbiological safety by the manufacturer prior to use. The silkworm pills used in this study were developed specifically for research purposes. The silkworm pills contained 2.5 g of silkworms and provided 10 kcal per daily dose, whereas the placebo pills contained 2.5 g of dextrin and provided 9.5 kcal (Supplementary Table 2). To maintain their stability, participants were instructed to store the ONS and pills at room temperature in a shaded environment throughout the study period.

### 2.3 Study protocols

The nutritional intervention program was conducted at the outpatient clinic of the Department of Neurology, Ajou University Hospital, for 12 weeks by a research nurse and licensed dietitian. Participants were randomly assigned in a 1:1 ratio to the placebo or ONS plus silkworm pill groups using a block randomization procedure. Participants allocated to the placebo group consumed placebo pills and ONS, whereas those assigned to the ONS plus silkworm pill group consumed silkworm pills and commercial ONS. This study was conducted in a double-blind manner. Both the researchers and the participants were blinded to their group assignments (i.e., whether they were allocated to the placebo or control group). Blinding was conducted by Maeil Inc. which generated the allocation using a block randomization schedule with a 2 × 2 allocation key and provided the assignments to the investigators. Maeil Inc. also delivered the study products in three separate shipments. The first shipment was sent to the investigators, who then distributed a 1-week supply to each participant at their initial visit. The second shipment consisted of a 4-week supply that was delivered directly to each participant's home. The final shipment, delivered in the fifth week, provided the remaining supply for the study period. These products were labeled with codes that prevented any identification of the assigned group, thus ensuring the double-blind integrity throughout the study. All participants were provided comprehensive information on the nutritional content of the product, recommended daily intake, method of consumption, and associated precautions, including restrictions associated with taking a new dietary supplement. During the 12-week study period, participants were instructed to consume the allotted pills (2.5 g) and ONS (200 ml) daily as a snack between meals. Throughout the 12-week intervention period, the research team contacted participants weekly by phone or message to monitor concomitant medication use and to confirm and assess compliance with the intake of ONS and silkworm pills. The recommended intake time was between meals as a snack, especially between breakfast and lunch or between lunch and dinner.

In addition, nutritional education was provided to both groups with the same content four times during the study period: at Weeks 0, 3, 6, and 9. The education consisted of four topics, each presented in a different format, including the proven effectiveness of the MIND diet in dementia prevention. The Week 0 session was delivered in person, while the sessions at Weeks 3, 6, and 9 were conducted as 10-min phone consultations. At Week 6, a nutritional evaluation was conducted via phone without an in-person visit, comprising 24-h dietary recalls, MIND diet adherence assessment, and either the Nutrition Quotient (NQ) or the Nutrition Quotient for the Elderly (NQ-E), depending on the participant's age group. Participants attended study visits at Week 0 (baseline) and Week 12 (end of intervention), during which evaluations and assessments were conducted.

**Figure 1:**
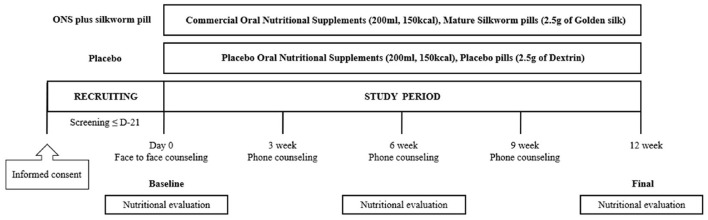
Flow chart of the study protocol. ONS, oral nutritional supplements.

The protocol and flow of the nutritional intervention are shown in Figure 1.

### 2.4 Outcome measures

Before and after the 12-week intervention, cognitive function was evaluated using the Korean version of the Mini-Mental Status Examination (K-MMSE) Clinical Dementia Rating (CDR), and Korean version of the Repeatable Battery for the Assessment of Neuropsychological Status (K-RBANS) (14, 19). Nutritional indicators were evaluated using a nutritional questionnaire, blood analysis, and dietary assessment. The nutritional questionnaire included the Mediterranean DASH Intervention Diet for Delaying Neurodegenerative Disease (MIND) score and nutritional quotient (NQ) or NQ-Elderly scores (6, 20). Blood sample analyses included total cholesterol, triglyceride, high-density lipoprotein (HDL), low-density lipoprotein (LDL), and C-reactive protein (CRP) levels and nutrients such as vitamin C, vitamin B12, and folic acid. At baseline and after the completion of the 12-week intervention, 30 cc of blood was collected from each participant. Fasting was maintained for 8 h before blood collection. The baseline period was from 21 days before to 1 day after study enrollment, and after completing the 12-week intervention, blood was collected no later than 14 days after completion. Blood analysis was conducted by the Department of Laboratory Medicine at Ajou University Hospital, employing distinct methodologies: Vitamin C, vitamin B12 and folic acid were assessed via electrochemiluminescence immunoassay (ECLIA); the lipid panel, encompassing total cholesterol, triglyceride, HDL and LDL, utilized an enzymatic colorimetric assay; and CRP was determined through the particle enhanced immunoturbidimetric assay (PETIA). Dietary intake was assessed at both baseline and after the 12-week intervention using the 24-h dietary recall method, with data collected before and after participants received dietary education. This procedure reviewed the dietary composition and overall intake of calories, proteins, carbohydrates, lipids, vitamins, and minerals. For the dietary analysis, only nutrients consumed through regular food intake were included, while those provided by the intervention products (ONS and silkworm pill) were excluded. The aim was to evaluate participants' habitual dietary intake and identify any significant changes in nutrient intake independent of the intervention. This approach allowed us to verify that the observed effects were attributable to the intervention itself rather than to changes in existing dietary habits. Our analysis showed no significant differences in caloric or nutrient intake before and after the intervention, further suggesting that the nutritional intervention did not substantially influence participants' usual diet and minimizing potential recall bias.

### 2.5 Statistical analysis

#### 2.5.1 Sample size calculation

Based on the literature review data, the mean values for the change in RBANS immediate recall after the 12-week intervention were assumed to be 3.1 ± 13.1 and −4.2 ± 11.6, respectively (21). The sample size was obtained using a two-sided test significance level of 0.05 and a power of 80% for comparison of means of RBANS immediate recall, which is the primary biomarker. Based on the calculations, the required sample size per group was determined to be 47. Considering a dropout rate of 10%, the final number of participants per group was calculated as 53, for a total of 106 participants (using PASS software version 12.0).

#### 2.5.2 Analysis of measured results

Only participants who completed all the follow-up assessments were analyzed (*n* = 62). Demographic and clinical characteristics of the participants were summarized using descriptive statistics. The normality of the data was tested using the Shapiro–Wilk test at a 5% significance level. Differences between groups were assessed using the independent *t*-test or Mann–Whitney *U*-test for continuous variables, and the chi-square test for discrete variables. Changes from baseline to 12 weeks were analyzed using the paired *t*-test or Wilcoxon signed-rank test.

Statistical analyses were performed using SPSS 26.0 (IBM Corp., Armonk, NY). A *p-*value < 0.05 was considered statistically significant.

### 2.6 Ethic statement

This study was approved by the institutional review board of the Ajou University Hospital (approval number: AJOUIRB-FOD-2021-712). All participants provided written informed consent before enrollment in the study (Clinical Research Information Service of Korea registration number: KCT0007304).

## 3 Results

### 3.1 Demographic and clinical characteristics of participants

A total of 109 participants were recruited and assessed for eligibility; however, 25 withdrew during the screening process. Consequently, 84 participants were randomly assigned to the control (*n* = 43) and silkworm groups (*n* = 41) in a 1:1 ratio. During the 12-week intervention period, four participants in the placebo group and four in the ONS plus silkworm pill group discontinued the intervention due to adverse events, including diarrhea, abdominal distension, and indigestion. As a result, 39 participants (90.7%) in the control group and 37 participants (90.2%) in the ONS plus silkworm pill group completed the intervention program. During the subsequent data analysis phase, an additional seven participants from each group were excluded from the final statistical analysis due to issues such as inadequate compliance and incomplete data records. Therefore, the final analyses were conducted on data from 30 participants in the placebo group and 32 participants in the ONS plus silkworm pill group. Due to practical constraints related to the investigational products' shelf-life of ~1 year, extending participant enrollment beyond this period would have necessitated producing a new batch. This raised concerns about potential batch-to-batch variability and product consistency. Consequently, all researchers reached a consensus during the study—agreed upon by both the investigators and the sponsoring company—to conduct an interim analysis. This analysis was carried out by the research team, with its procedures and results reviewed in consultation with an independent statistical expert to ensure the validity of the findings. As the interim statistical analysis demonstrated a statistically significant finding for the primary marker at that point, the study was subsequently terminated by the researchers and did not recruited participants.

**Figure 2:**
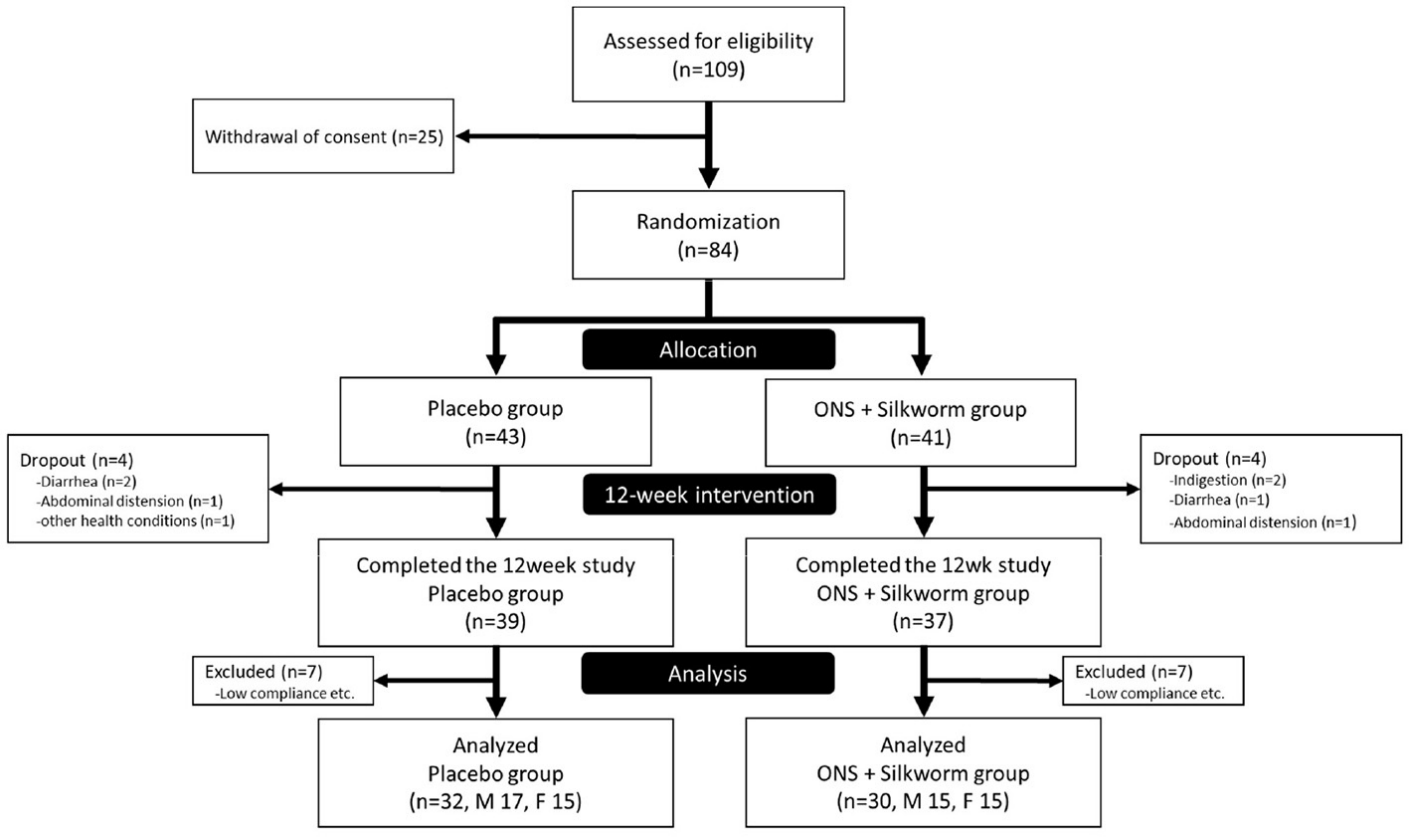
Flowchart of the participants.

Among these, 14 participants were excluded due to < 70% compliance, and thus results were analyzed using data from 62 participants, including 32 in the placebo group and 30 in the silkworm group (Figure 2).

Table 1 shows the demographic and baseline clinical characteristics of the study participants. The mean age was 70.6 years and 48.4% of participants were female. The average number of years of education was 11.7 years, and the mean K-MMSE score and clinical dementia rating (CDR) were 25.1 and 0.5, respectively. There were no significant differences in the cognitive and nutritional variables or demographic characteristics between the placebo and ONS plus silkworm pill groups.

**Table 1:** Demographic and baseline clinical characteristics of participants.

**Characteristics**	**Total (*n* = 62)**	**Placebo group^a^ (*n* = 32)**	**ONS plus silkworm pill^b^ (*n* = 30)**	***p*-Value^c^**
Age (years)	70.6 ± 8.5^b^	70.3 ± 8.5	70.9 ± 8.6	0.261
Sex, female (*n*, %)	30, 48.4%	15, 46.9%	15, 50%	0.806
BMI (kg/m^2^)	24.0 ± 2.9	24.0 ± 3.1	24.0 ± 2.88	0.475
Education (years)	11.7 ± 3.7	12.1 ± 3.2	11.4 ± 4.3	0.430
**Medical history**				
Hypertension (*n*, %)	24, 38.7%	11, 34.4%	13, 43.3%	0.469
Diabetes mellitus (*n*, %)	12, 19.4%	6, 18.8%	6, 20%	0.901
Dyslipidemia (*n*, %)	14, 22.6%	9, 28.1%	5, 16.7%	0.281
Coronary disease (*n*, %)	7, 11.3%	1, 3.1%	6, 20%	0.360
Liver disease (*n*, %)	1, 1.6%	1, 3.1%	0, 0%	0.329
Kidney disease (*n*, %)	2, 3.2%	0, 0%	2, 6.7%	0.138
Others (*n*, %)	7, 11.3%	5, 15.6%	2, 6.7%	0.265
**Cognition related medication use**				
Acetylcholinesterase inhibitors (*n*, %)	41, 66.1%	20, 62.5%	21, 70.0%	0.841
Cognitive enhancer/supplements (*n*, %)	42, 67.6%	23, 71.9%	19, 63.3%	0.622
None	5, 8.1%	2, 6.3%	3, 10.0%	0.171
**Cognition**				
K-MMSE total score	25.1 ± 3.0	25.4 ± 3.1	24.7 ± 3.0	0.304
CDR	0.5 ± 0.0	0.5 ± 0.0	0.5 ± 0.0	0.340
CDR-SOB	1.3 ± 0.9	1.3 ± 0.9	1.3 ± 0.9	0.988
K-IADL	0.2 ± 0.1	0.2 ± 0.1	0.2 ± 0.1	0.571
K-RBANS, total	75.4 ± 18.9	78.7 ± 17.5	73.5 ± 21.5	0.686
K-RBANS, immediate recall	78.3 ± 14.1	80.6 ± 13.2	76.6 ± 15.4	0.844
K-RBANS, delayed recall	72.5 ± 18.9	77.8 ± 19.3	66.0 ± 18.9	0.326
K-RBANS, language	87.7 ± 16.1	87.7 ± 16.8	87.8 ± 16.1	0.929
K-RBANS, visuospatial	78.5 ± 17.4	80.4 ± 17.0	77.3 ± 19.6	0.842
K-RBANS, attention	100.4 ± 16.2	99.9 ± 11.8	102.5 ± 19.7	0.164
**Nutrition**				
MIND score	6.1 ± 1.3	6.1 ± 1.3	6.2 ± 1.2	0.912
Total cholesterol (mg/dl)	181.0 ± 38.7	184.1 ± 40.5	177.7 ± 37.8	0.181
Triglyceride (mg/dl)	102.9 ± 39.8	114.5 ± 51.1	91.1 ± 28.4	0.488
HDL (mg/dl)	58.9 ± 14.2	58.1 ± 14.3	59.7 ± 14.8	0.911
LDL (mg/dl)	101.5 ± 34.3	103.1 ± 37.5	99.8 ± 33.0	0.284
CRP (mg/dl)	0.1 ± 0.1	0.1 ± 0.1	0.1 ± 0.1	0.326
Folic acid (ng/ml)	15.9 ± 9.3	17.0 ± 10.3	15.0 ± 8.0	0.746
Vit. B_12_ (pmol/L)	894.6 ± 523.2	915.5 ± 459.1	874.0 ± 609.3	0.812

^a^Placebo, dextrin pills, and placebo ONS containing carbohydrates, proteins, and lipids.

^b^Silkworm, silkworm pills, and commercial ONS.

^c^Significantly different at p < 0.05, by the independent t-test and chi-square test.

BMI, body mass index; CDR, clinical dementia rating; CDR-SOB, clinical dementia rating sum of boxes; CRP, C-reactive protein; HDL, high-density lipoprotein; K-IADL, Korean instrumental activities of daily living; K-MMSE, Korean version of the mini-mental status examination; LDL, low-density lipoprotein; MIND, mediterranean-DASH intervention for neurodegenerative delay; K-RBANS, Korean version of the repeatable battery for the assessment of neuropsychological status; Vit., vitamin.

### 3.2 Changes in cognitive function after the 12-week nutritional intervention

After 12 weeks of intervention, the delayed memory domain score of the K-RBANS showed improvement in the ONS plus silkworm pill group (65.9 ± 19.0 vs. 70.5 ± 19.0, *p* = 0.011), and there was also a significant difference between the placebo and ONS plus silkworm pill groups. The results demonstrate a statistically significant difference in the change between the placebo and test groups (*p* = 0.018). The mean difference in change was 5.4 units, with a 95% confidence interval ranging from 1.0 to 9.8. This interval excludes zero, indicating that the observed difference is unlikely to be due to chance alone. The corresponding *F*-value was ~6.0, representing a moderate effect size (η^2^ = 0.09). The immediate memory domain score of the K-RBANS improved in the placebo group (80.6 ± 13.2 vs. 85.7 ± 14.3, *p* = 0.001); however, there was no significant difference between the placebo and ONS plus silkworm pill groups. There were no significant differences in the total scale, language, visuospatial, attention scores of the K-RBANS, K-MMSE total score, and CDR. No notable differences were observed between the two groups before and after the intervention (Table 2).

**Table 2:** Changes in cognitive function after the 12-week study period.

**Cognitive function test**	**Placebo group**^**a**^ **(*****n*** = **32)**			**ONS plus silkworm pill group**^**b**^ **(*****n*** = **30)**			***p*-Value^d^**
	**0 week**	**12 week**	***p*****-Value**^c^ Δ**12 w**−**0 w**	**0 week**	**12 week**	***p*****-Value**^c^ Δ**12 w**−**0 w**	
K-RBANS total scale	78.7 ± 17.5	78.7 ± 17.0	1.000	73.5 ± 21.5	74.8 ± 21.9	0.307	0.854^†^
K-RBANS immediate memory	80.6 ± 13.2	85.7 ± 14.3	0.001^**^	76.6 ± 15.4	77.8 ± 15.7	0.420	0.070
K-RBANS delayed memory	77.8 ± 19.3	77.0 ± 20.6	0.566	65.9 ± 19.0	70.5 ± 19.0	0.011^††^	0.018^*^
K-RBANS language	87.7 ± 16.8	87.2 ± 16.0	0.815	87.8 ± 16.1	86.2 ± 18.5	0.496	0.711
K-RBANS visuospatial	80.4 ± 17.0	78.2 ± 14.9	0.272	77.3 ± 19.6	79.7 ± 18.5	0.182	0.088
K-RBANS attention	99.9 ± 11.8	98.4 ± 13.7	0.214^†^	102.5 ± 19.7	99.4 ± 18.8	0.090	0.475
K-MMSE total score	25.4 ± 3.1	25.4 ± 2.7	1.141	24.7 ± 3.0	24.8 ± 3.4	0.868	0.289
CDR	0.5 ± 0.0	0.5 ± 0.0	1.000	0.5 ± 0.0	0.5 ± 0.1	0.317	0.302
CDR-SOB	1.3 ± 0.9	1.3 ± 1.0	0.366	1.3 ± 0.9	1.3 ± 1.1	0.378	0.170

^a^Placebo, dextrin pills, and placebo ONS containing carbohydrates, proteins, and lipids.

^b^Silkworm, silkworm pills, and commercial ONS.

^c^Significance as determined by a paired t-test. ^*^p < 0.05, ^**^p < 0.01, ^†^Significance as determined by Wilcoxon signed-rank test. ^††^*p* < 0.05.

^d^Significance as determined by the independent t-test. ^*^p < 0.05, ^†^Significance as determined by the Mann–Whitney U-test.

CDR, clinical dementia rating, CDR-SOB, clinical dementia rating sum of boxes; K-MMSE, Korean version of the mini-mental status examination; K-RBANS, Korean version of the repeatable battery for the assessment of neuropsychological status.

### 3.3 Changes in nutritional factors after the 12-week nutritional intervention

Changes in nutritional markers after 12 weeks of intervention are shown in Table 3. The MIND score was improved in both groups (placebo group: 6.1 ± 1.3 vs. 6.9 ± 1.0, *p* = 0.005, effect size = 0.69; ONS plus silkworm pill group: 6.2 ± 1.2 vs. 7.1 ± 1.1, *p* = 0.003, effect size = 0.78). Improvement was also observed in the NQ total score in both groups (placebo group: 68.5 ± 13.0 vs. 74.6 ± 10.9, *p* = 0.002, effect size = 0.51; ONS plus silkworm pill group: 68.5 ± 12.5 vs. 74.9 ± 10.8, effect size = 0.001, *d* = 0.55). However, there were no significant differences in either the MIND score or NQ total score between the two groups before and after the intervention. In the placebo group, LDL levels decreased significantly (103.1 ± 37.5 mg/dl vs. 95.2 ± 35.8 mg/dl, *p* = 0.025, effect size = −0.22). In the ONS plus silkworm pill group, vitamin B_12_ levels increased markedly (874.0 ± 609.3 pmol/L vs. 1,083.5 ± 765.2 pmol/L, *p* = 0.001, effect size = 0.30). However, there were no significant differences in other blood measurement results before and after the 12-week intervention within each group, as well as in the pre- and post-comparison results between the two groups.

**Table 3:** Changes in nutritional markers after the 12-week study period.

**Nutritional marker**	**Placebo group**^**a**^ **(*****n*** = **32)**			**ONS plus silkworm pill group**^**b**^ **(*****n*** = **30)**			***p*-Value^d^**
	**0 week**	**12 week**	***p*****-Value**^c^ Δ**12 w**−**0 w**	**0 week**	**12 week**	***p*****-Value**^c^ Δ**12 w**−**0 w**	
MIND score	6.1 ± 1.3	6.9 ± 1.0	0.005^**^	6.2 ± 1.2	7.1 ± 1.1	0.003^**^	0.818
NQ (total)	68.5 ± 13.0	74.6 ± 10.9	0.002^††^	68.5 ± 12.5	74.9 ± 10.8	< 0.001^††^	0.783
Total cholesterol (mg/dl)	184.1 ± 40.5	177.3 ± 38.5	0.136	177.7 ± 37.8	177.0 ± 42.1	0.816	0.277
Triglyceride (mg/dl)	114.5 ± 51.1	110.7 ± 40.6	0.751^†^	91.1 ± 28.4	107.8 ± 64.7	0.309^†^	0.379^†^
HDL (mg/dl)	58.1 ± 14.3	60.0 ± 14.0	0.289^†^	59.7 ± 14.8	59.8 ± 13.8	0.949	0.612^†^
LDL (mg/dl)	103.1 ± 37.5	95.2 ± 35.8	0.025^*^	99.8 ± 33.0	95.8 ± 37.0	0.246	0.421
CRP (mg/dl)	0.1 ± 0.1	0.1 ± 0.1	0.673^†^	0.1 ± 0.1	0.2 ± 0.6	0.129^†^	0.163^†^
Folic acid (ng/ml)	17.0 ± 10.3	17.7 ± 11.8	0.854^†^	15.0 ± 8.0	17.4 ± 9.6	0.071^†^	0.193^†^
Vit. B_12_ (pmol/L)	915.5 ± 459.1	1,060.7 ± 829.6	0.063^†^	874.0 ± 609.3	1,083.5 ± 765.2	< 0.001^††^	0.159^†^

^a^Placebo, dextrin pills, and placebo ONS containing carbohydrates, proteins, and lipids.

^b^Silkworm, silkworm pills, and commercial ONS.

^c^Significance as determined by a paired t-test. ^*^p < 0.05, ^**^p < 0.01, ^†^Significance as determined by the Wilcoxon signed-rank test. ^††^*p* < 0.005.

^d^Significance as determined by the independent t-test. ^*^p < 0.05, ^†^Significance as determined by the Mann–Whitney U-test. ^††^*p* < 0.05.

CRP, C-reactive protein; HDL, high-density lipoprotein; LDL, low-density lipoprotein; MIND, mediterranean-DASH intervention for neurodegenerative delay; NQ, nutrition quotient, Vit., vitamin.

Table 4 presents changes in the dietary intake before and after the 12-week intervention. Only nutrients consumed through food were included, and nutrients consumed through silkworm pills and commercial ONS or other nutritional supplements were excluded. In the placebo group, there were no significant differences between weeks 0 and 12, both within and between groups. In the ONS plus silkworm pill group, zinc intake was significantly decreased (11.8 ± 5.0 mg vs. 9.4 ± 4.7 mg, *p* = 0.049). There was also a significant difference in zinc intake between the placebo and silkworm pill groups (*p* = 0.049); however, for other nutrients, there was no notable differences within the groups at weeks 0 and 12, as well as in the pre- and post-comparison results between the two groups.

**Table 4:** Changes in nutrients intake assessed by food diary after 12-week study period.

**Nutrients**	**Placebo group**^**a**^ **(*****n*** = **32)**			**ONS plus silkworm pill group**^**b**^ **(*****n*** = **30)**			***p*-Value^e^**
	**0 week**	**12 week**	***p*****-Value**^c^ Δ**12 w**−**0 w**	**0 week**	**12 week**	***p*****-Value**^d^ Δ**12 w**−**0 w**	
Energy (kcal)	2,058.2 ± 568.1^a^	1,990.3 ± 625.9	0.654	1,938.9 ± 601.2	1,668.1 ± 599.3	0.063	0.226
Carbohydrate (g)	280.0 ± 82.1	267.5 ± 73.5	0.640	267.7 ± 79.6	235.8 ± 108.2	0.030^†^	0.226
Fat (g)	60.9 ± 25.7	62.0 ± 31.5	0.881	57.0 ± 30.3	50.0 ± 25.9	0.478	0.499
Protein (g)	83.8 ± 33.2	86.6 ± 37.6	0.667	86.3 ± 36.6	68.4 ± 32.1	0.057	0.063
Vitamin A (μg RAE)	494.5 ± 263.2	578.6 ± 651.3	0.926	586.1 ± 506.5	471.2 ± 301.7	0.644	0.822
Vitamin D (μg)	3.3 ± 4.0	5.0 ± 9.3	0.766	7.2 ± 10.7	5.2 ± 8.0	0.471	0.545
Vitamin E (mg)	18.4 ± 10.3	21.3 ± 12.5	0.525	18.0 ± 10.1	15.7 ± 7.1	0.289	0.105
Vitamin K (μg)	241.9 ± 199.1	226.2 ± 174.7	0.911	175.0 ± 142.4	226.7 ± 192.8	0.185	0.499
Vitamin C (mg)	156.9 ± 122.6	176.5 ± 133.8	0.588	133.3 ± 94.5	141.4 ± 91.2	0.926	0.683
Vitamin B_1_ (mg)	2.1 ± 1.4	2.1 ± 1.5	0.866	1.9 ± 0.9	1.6 ± 0.7	0.131	0.2
Vitamin B_2_ (mg)	1.8 ± 1.5	1.8 ± 1.6	0.881	1.7 ± 0.7	1.5 ± 0.8	0.517	0.508
Vitamin B_3_ (mg)	14.8 ± 7.0	15.6 ± 7.4	0.501	16.6 ± 6.8	14.4 ± 7.2	0.192	0.163
Vitamin B_6_ (mg)	2.3 ± 2.2	4.3 ± 9.3	0.313	1.8 ± 0.6	1.6 ± 0.7	0.125	0.102
Folic acid (μg)	573.8 ± 229.1	620.5 ± 415.2	0.695	555.6 ± 218.5	555.3 ± 267.1	0.719	0.714
Vitamin B_12_ (μg)	9.5 ± 8.0	11.5 ± 7.6	0.331	12.3 ± 12.3	12.6 ± 11.5	0.813	0.398
Calcium (mg)	643.9 ± 370.3	664.3 ± 334.8	0.550	569.9 ± 261.6	605.1 ± 294.7	0.393	0.866
Magnesium (mg)	121.0 ± 85.4	127.3 ± 94.5	0.765	114.8 ± 71.8	116.9 ± 76.7	0.975	0.866
Iron (mg)	18.4 ± 7.1	19.4 ± 11.5	0.940	20.2 ± 9.5	16.4 ± 9.0	0.054	0.081
Zinc (mg)	11.0 ± 4.5	13.5 ± 10.6	0.313	11.8 ± 5.0	9.4 ± 4.7	0.049^†^	0.049^†^
Selenium (μg)	70.9 ± 48.0	76.8 ± 43.7	0.765	86.0 ± 53.4	68.4 ± 47.5	0.131	0.237

^a^Placebo, dextrin pills, and placebo ONS containing carbohydrates, proteins, and lipids.

^b^Silkworm, silkworm pills, and commercial ONS.

^c^Significance as determined by the Wilcoxon signed-rank test. ^†^*p* < 0.05.

^d^Significance as determined by the Mann-Whitney U-test. ^†^*p* < 0.05.

^e^Significance as determined by Mann-Whitney U-test. ^†^*p* < 0.05.

## 4 Discussion

In this study, we demonstrated that providing mature silkworm pills made from golden silkworm, combined with commercial ONS, to individuals aged ≥50 years diagnosed with MCI for 12 weeks had a positive effect on improving delayed memory recall compared with the control group. Nutrition-related assessment results (NQ and the MIND score) were significantly improved in both the experimental and control groups, with no significant differences between the groups. These results suggest that the combination of mature silkworm pills and commercial ONS may improve cognitive performance in patients with MCI. Additionally, we confirmed that nutritional education can improve the MIND dietary compliance and eating habits in these patients.

Previous clinical studies have established positive effects on pathophysiological mechanisms by investigating the proliferative effects of mature silkworms on nerve cells and cognitive enhancement (22–25). In previous animal experiments, scopolamine-induced reversible short-term memory loss in rodents was attenuated following golden silkworm administration. These studies reported that golden silkworm reduced oxidative stress, increased mitochondrial respiratory complex activity in the brain, elevated ATP levels, and inhibited neuronal degeneration and memory loss (17). Studies have shown that extracts derived from *Bombyx mori*, including BF-7 and mature silkworm powder, improve memory and learning in human and animal models through multiple mechanisms such as enhancing acetylcholine levels, protecting against oxidative stress, and supporting mitochondrial function (19, 26, 27). These findings suggest potential neuroprotective roles in age-related cognitive decline and neurodegenerative disease models, including Alzheimer's and Parkinson's disease.

However, subsequent clinical evidence is limited, because previous studies have primarily examined the effects of mature silkworms in isolation. Our study extends these findings by demonstrating clinically significant benefits when evaluating the combined effects of mature silkworms combined with essential nutrients known to be deficient in dementia patients, including vitamin B_12_, folic acid, vitamin C, vitamin E, selenium, and phospholipids (28).

In this study, the significant improvement in the delayed memory index is consistent with the results from a previous study that showed the beneficial effects of ONS (Souvenaid, Nutricia), specially designed for management of early dementia, for 12 weeks on cognitive function in patients with mild dementia using the revised version of Wechsler Memory Scale (9). In that study, they used a special formula of multinutrient combination Fortasyn Connect, which is not included in our study. While the control group showed neither deterioration nor improvement in cognitive function, the experimental group showed improved delayed verbal recall scores after 12 weeks. These findings suggested that specially designed ONS may support delayed memory function in dementia patients. An interim analysis revealed statistically significant improvement in the primary outcome measure, prompting early study termination and cessation of further participant enrollment.

Similarly, our study indicated that supplementation with mature silkworms and commercial standard ONS can enhance the delayed memory index in the K-RBANS assessment, as evidenced by significant increases both within and between groups under the experimental conditions. The K-RBANS comprises the immediate memory index, visuospatial/construction ability, language ability, attention, delayed memory, and total score (29). Among these indicators, the delayed memory test is particularly sensitive to early memory impairment compared with other measures (30), and it can effectively detect early dementia by assessing anterograde memory capacity (14). Therefore, the significant increase in the delayed memory index observed in our study can be interpreted as an improvement in participants' anterograde memory function compared with baseline.

Additionally, during the intervention period, participants received four sessions of the MIND diet and dietary habit nutrition education, resulting in significantly increased NQ scores in both groups. These results are consistent with those of the previous SUPERBRAIN study, in which increased NQ scores following the MIND nutrition education were attributed to improved dietary habits and nutritional status among participants (6). Similarly, the positive changes in both the MIND checklist and NQ scores in our study can be interpreted as successful nutritional intervention outcomes, indicating improvements in dietary habits and meal quality in patients with MCI. Despite improvements in dietary habits and dietary quality as assessed by NQ, no significant difference was observed in the total K-RBANS score. This outcome may be influenced by factors such as medication use, the progression of MCI, and nutrient intake. Notably, 66.1% of patients with MCI in this study were on dementia-related medications, acetylcholinesterase inhibitors and those not on dementia-specific medications were using cognitive enhancers. Only five of the 26 participants (8.1%) were not taking any cognition related medications. Consequently, these patients were in a more active cognitive state than those in previous studies that focused on MCI. The MIND diet is designed to prevent cognitive decline in older adults rather than to treat MCI or dementia (31), which may have limited its effects on advanced patients receiving cognitive function medications. Consistently, a previous study found that ONS (Souvenaid) did not benefit patients with mild-to-moderate Alzheimer's who were already on medication. In that study, 527 participants received ONS for 24 weeks; however, no delay in cognitive decline was observed using the ADAS-cog assessments (7). Furthermore, nutrient intake analysis revealed a significant decrease in zinc intake within the experimental group, with significant differences between the groups. This suggests that the nutrient intake of the experimental group was insufficient compared with that of the control group, potentially negatively affecting the study results (32). Despite this, among the nutritional blood indicators (TC, TG, HDL, LDL, CRP, folic acid, and vitamin B12), significant improvement was observed only in vitamin B12 levels within the experimental group. This suggests that supplementation with mature silkworm pills and commercial ONS for over 12 weeks positively contributes to nutrient intake, which supports cognitive function.

Although these findings are beneficial, they have some limitations. First, further research is required to determine the appropriate dosage for mature silkworms, as no human studies have specifically identified the most effective dosage. The dosage should vary based on age, sex, and physiological condition, with recommendations ranging from 1 to 500 mg/kg/day (33). In this study, assuming an average body weight of 60 kg, 2.5 g dosage corresponds to ~41.7 mg/kg/day, which falls within the previously reported range. This indicates the need for further research with larger quantities of mature silkworms. Second, the 12-week intervention period may have been insufficient to observe significant cognitive improvement. A previous study involving 311 patients with MCI extended their participation for up to 72 months, following a 24-month intervention. The results indicated that prolonged use of ONS m (Souvenaid) delayed the decline in cognition, function, brain atrophy, and disease progression, suggesting that longer intervention periods improve cognitive function in patients with MCI (34). Future research should consider extending the intervention period and performing subsequent evaluations to identify the optimal timing. Third, this study was terminated early following an interim analysis that demonstrated statistical significance in the predefined primary outcome. The timing of the interim analysis was aligned with the 1-year shelf-life of the investigational products. Extending recruitment beyond this point would have required the manufacture of a new batch, which could have introduced potential variability in product consistency. To avoid this issue, the investigators and sponsoring company had agreed in advance that the trial would be discontinued if statistical significance was observed at the 1-year interim analysis. While the interim analysis was conducted by the research team and reviewed in consultation with a statistical expert, we acknowledge that stopping the study early based on observed significance may increase the risk of Type I error and overestimation of the treatment effect. This limitation should be considered when interpreting the findings. Lastly, a personal and family history of disease and drug survey revealed a higher use of heart disease medication in the experimental group (20%) than in the control group (3.1%). This indicated that the experimental group had more health risk factors because cardiovascular diseases and heart conditions are strongly associated with an increased risk of MCI and dementia (26). These factors may have negatively affected our results. Future research should adjust for diseases and medication histories that could affect the outcomes and implement random allocation. Furthermore, mature silkworm powder contains various bioactive compounds such as peptides, polyphenols, and flavonoids, which have been reported to exert antioxidant, anti-inflammatory, and neuroprotective effects (11–13, 27). These properties may contribute to its potential for cognitive improvement.

Despite these limitations, this study had several strengths. To the best of our knowledge, this is the first randomized, double-blind, controlled human trial aimed at improving cognitive function in patients with MCI through supplementation with mature silkworms and commercial ONS, suggesting the possibility of developing special foods for medical purposes, specifically targeting the older population at risk of dementia. The 12-week hospital-based intervention was meticulously controlled with the active involvement of medical staff, including doctors, nurses, and dietitians, allowing for immediate reporting and management of any adverse effects or drug interactions.

In conclusion, this study demonstrated that a 12-week supplementation with mature silkworm powder and commercial ONS led to improvements in cognitive function and enhanced the delayed memory index in patients with MCI. Further clinical trials are warranted to explore the efficacy of this intervention in patients with MCI and dementia. Administering mature silkworm pills and commercial ONS to patients who are not on medication and to those with mild dementia who are on medication is justified to confirm and expand upon these findings. Future clinical trials should aim to measure relevant biomarkers to substantiate these results, which suggest that mature silkworms and nutritional supplements can aid in improving cognitive function.

## Data Availability

The original contributions presented in the study are included in the article/Supplementary material, further inquiries can be directed to the corresponding authors.

## References

[1] Ngandu T Lehtisalo J Solomon A Levalahti E Ahtiluoto S Antikainen R. A 2 year multidomain intervention of diet, exercise, cognitive training, and vascular risk monitoring versus control to prevent cognitive decline in at-risk elderly people (FINGER): a randomised controlled trial. Lancet. (2015) 385:2255–63. 10.1016/S0140-6736(15)60461-5 25771249

[2] Kivipelto M Mangialasche F Snyder HM Allegri R Andrieu S Arai H. World-wide FINGERS network: a global approach to risk reduction and prevention of dementia. Alzheimers Dement. (2020) 16:1078–94. 10.1002/alz.12123 32627328 PMC9527644

[3] Park HK Jeong JH Moon SY Park YK Hong CH Na HR. South Korean study to prevent cognitive impairment and protect brain health through lifestyle intervention in at-risk elderly people: protocol of a multicenter, randomized controlled feasibility trial. J Clin Neurol. (2020) 16:292. 10.3988/jcn.2020.16.2.292 32319247 PMC7174118

[4] Moon SY Kim S Choi SH Hong CH Park YK Na HR. Impact of multidomain lifestyle intervention on cerebral cortical thickness and serum brain-derived neurotrophic factor: the SUPERBRAIN exploratory sub-study. Neurotherapeutics. (2022) 19:1514–25. 10.1007/s13311-022-01276-x 35915368 PMC9606175

[5] Lehtisalo J Ngandu T Valve P Antikainen R Laatikainen T Strandberg T. Nutrient intake and dietary changes during a 2-year multi-domain lifestyle intervention among older adults: secondary analysis of the Finnish geriatric intervention study to prevent cognitive impairment and disability (FINGER) randomised controlled trial. Br J Nutr. (2017) 118:291–302. 10.1017/S0007114517001982 28875868

[6] Park YK Song JE Hong CH Moon SY Jeong JH Park HK. SoUth Korean study to PrEvent cognitive impaiRment and protect BRAIN (SUPERBRAIN) showed improvement in cognitive function mostly in individuals with nutritional improvement: sub-analysis of SUPERBRAIN results. Alzheimers Dement. (2021) 17:e054727. 10.1002/alz.054727

[7] Shah RC Kamphuis PJ Leurgans S Swinkels SH Sadowsky CH Bongers A. The S-connect study: results from a randomized, controlled trial of Souvenaid in mild-to-moderate Alzheimer's disease. Alzheimers Res Ther. (2013) 5:1–9. 10.1186/alzrt224 24280255 PMC3978853

[8] Scheltens P Twisk JW Blesa R Scarpini E von Arnim CA Bongers A. Efficacy of Souvenaid in mild Alzheimer's disease: results from a randomized, controlled trial. J Alzheimers Dis. (2012) 31:225–36. 10.3233/JAD-2012-121189 22766770

[9] Scheltens P Kamphuis PJ Verhey FR Rikkert MGO Wurtman RJ Wilkinson D. Efficacy of a medical food in mild Alzheimer's disease: a randomized, controlled trial. Alzheimers Dement. (2010) 6:1–10.e1. 10.1016/j.jalz.2009.10.003 20129316

[10] Onakpoya IJ Heneghan CJ. The efficacy of supplementation with the novel medical food, Souvenaid, in patients with Alzheimer's disease: a systematic review and meta-analysis of randomized clinical trials. Nutr Neurosci. (2017) 20:219–27. 10.1080/1028415X.2015.1110899 26638900

[11] Nguyen P Kim K-Y Kim A-Y Kang S Osabutey AF Jin H. The additive memory and healthspan enhancement effects by the combined treatment of mature silkworm powders and Korean angelica extracts. J Ethnopharmacol. (2021) 281:114520. 10.1016/j.jep.2021.114520 34391862

[12] Nguyen P Kim K-Y Kim AY Choi B-H Osabutey AF Park YH. Mature silkworm powders ameliorated scopolamine-induced amnesia by enhancing mitochondrial functions in the brains of mice. J Funct Foods. (2020) 67:103886. 10.1016/j.jff.2020.103886

[13] Ji S-D Kim S-B Kim K-Y Kim N-S Kim S-W Jo Y-Y. Contents of nutrients in ultra-fine powders of steamed and lyophilized mature silkworms generated by four silkworm varieties. J Asia Pac Entomol. (2019) 22:969–74. 10.1016/j.aspen.2019.07.009

[14] Park SJ Kim K-Y Baik M-Y Koh YH. Sericulture and the edible-insect industry can help humanity survive: insects are more than just bugs, food, or feed. Food Sci Biotechnol. (2022) 31:657–68. 10.1007/s10068-022-01090-3 35646418 PMC9133288

[15] Kim H-J Kim K-Y Ji S-D Lee H-T. Anti-melanogenic activity of steamed and freeze-dried mature silkworm powder. J Asia Pac Entomol. (2017) 20:1001–6. 10.1016/j.aspen.2017.07.013

[16] Cho J-M Kim K-Y Ji S-D Kim E-H. Protective effect of boiled and freeze-dried mature silkworm larval powder against diethylnitrosamine-induced hepatotoxicity in mice. J Cancer Prevent. (2016) 21:173. 10.15430/JCP.2016.21.3.173 27722143 PMC5051591

[17] Lee D-Y Cho J-M Yun S-M Hong K-S Ji S-D Son J-G. Comparative effect of silkworm powder from three *Bombyx mori* varieties on ethanol-induced gastric injury in rat model. Int J Indust Entomol. (2017) 35:1–6. 10.7852/ijie.2017.35.1.1

[18] Kim K-Y Koh Y-H. The past, present and future of silkworm as a natural health food. Food Sci Indust. (2022) 55:154–65. 10.23093/FSI.2022.55.2.154

[19] Kim K-Y Osabutey AF Nguyen P Kim S-B Jo Y-Y Kweon H. The experimental evidences of steamed and freeze-dried mature silkworm powder as the calorie restriction mimetics. Int J Ind Entomol Biomater. (2019) 39:511–20. 10.7852/ijie.2019.39.1.1

[20] Kang S-J Choi S-H Lee B-H Kwon J-C Na D-L Han S-H. The reliability and validity of the Korean Instrumental Activities of Daily Living (K-IADL). J Korean Neurol Assoc. (2002) 20:8–14.

[21] Kobayashi Y Kuhara T Oki M Xiao JZ. Effects of *Bifidobacterium breve* A1 on the cognitive function of older adults with memory complaints: a randomised, double-blind, placebo-controlled trial. Benef Microbes. (2019) 10:511–20. 10.3920/BM2018.0170 31090457

[22] Mai LX Kang SK Jo YY Nguyen P Kim AY Kim KY. An alkaline protease-digestion of silkworm powder enhances its effects over healthspan, autophagy, and mitochondria function in a rotenone-induced drosophila model. Front Nutr. (2022) 9:808295. 10.3389/fnut.2022.808295 35782921 PMC9244169

[23] Park J-O Koo B-H Kim J-Y Bai D-S Chang M-S Kim O-L. The Korean repeatable battery for the assessment of neuropsychological status-update: psychiatric and neurosurgery patient sample validity. J Korean Neurosurg Soc. (2021) 64:125–35. 10.3340/jkns.2020.0090 33105533 PMC7819793

[24] Yook S-M Lim Y-S Lee J-S Kim K-N Hwang H-J Kwon S. Revision of nutrition quotient for Korean adults: NQ-2021. J Nutr Health. (2022) 55:278–95. 10.4163/jnh.2022.55.2.278

[25] Randolph C Tierney MC Mohr E Chase TN. The repeatable battery for the assessment of neuropsychological status (RBANS): preliminary clinical validity. J Clin Exp Neuropsychol. (1998) 20:310–9. 10.1076/jcen.20.3.310.823 9845158

[26] Kim DK Kang YK Lee MY Lee K-G Yeo J-H Lee WB. Neuroprotection and enhancement of learning and memory by BF-7. J Health Sci. (2005) 51:317–24. 10.1248/jhs.51.317

[27] Nguyen P Kim K-Y Kim A-Y Kim N-S Kweon H Ji S-D. Increased healthspan and resistance to Parkinson's disease in Drosophila by boiled and freeze-dried mature silk worm larval powder. J Asia Pac Entomol. (2016) 19:551–61. 10.1016/j.aspen.2016.05.003

[28] Cardoso BR Cominetti C. Importance and management of micronutrient deficiencies in patients with Alzheimer's disease. Clin Interv Aging. (2013) 8:531–42. 10.2147/CIA.S27983 23696698 PMC3656646

[29] Dewar M Pesallaccia M Cowan N Provinciali L Della Sala S. Insights into spared memory capacity in amnestic MCI and Alzheimer's disease via minimal interference. Brain Cogn. (2012) 78:189–99. 10.1016/j.bandc.2011.12.005 22261228

[30] Morris MC Tangney CC Wang Y Sacks FM Barnes LL Bennett DA. MIND diet slows cognitive decline with aging. Alzheimers Dement. (2015) 11:1015–22. 10.1016/j.jalz.2015.04.011 26086182 PMC4581900

[31] Lee K-S Cheong H-K Oh B-H Hong C-H. Comparison of the validity of screening tests for dementia and mild cognitive impairment of the elderly in a community: K-MMSE, MMSE-K, MMSE-KC, and K-HDS. J Korean Neuropsychiatr Assoc. (2009) 48:61–9.

[32] Ji S-D Kim S-B Son J-G Kim S-W Jo Y-Y Kim J-G. Composition for Improving Memory or Composition for Preventing and Treating Alzheimer's Disease, Comprising Cooked Processed Silkworm Product Having Silk Protein. Korean Intellectual Property Office (2019).

[33] Soininen H Solomon A Visser PJ Hendrix SB Blennow K Kivipelto M. 36-month LipiDiDiet multinutrient clinical trial in prodromal Alzheimer's disease. Alzheimers Dement. (2021) 17:29–40. 10.1002/alz.12172 32920957 PMC7821311

[34] Wolters FJ Segufa RA Darweesh SK Bos D Ikram MA Sabayan B. Coronary heart disease, heart failure, and the risk of dementia: a systematic review and meta-analysis. Alzheimer Dement. (2018) 14:1493–504. 10.1016/j.jalz.2018.01.007 29494808

