# Supplementary material for: Effects of oral nutritional supplements plus mature silkworm pill on cognitive function in Korean adults with mild cognitive impairment: a 12-week randomized blinded clinical trial
Source: Front Nutr. 2025 Oct 28;12:1605525. doi: 10.3389/fnut.2025.1605525 (PMC12604101; doi:10.3389/fnut.2025.1605525)
Supplement: Supplementary file 1 [file Table_1.docx]

**Supplementary tables**

Supplementary table 1. Comparisons of nutritional composition between placebo ONS and commercial ONS

| **Nutrition Component** | **Placebo ONS** | | **Commercial ONS** | |
| --- | --- | --- | --- | --- |
|  | **Amount per daily dose** | **unit** | **Amount per daily dose** | **unit** |
| Energy (kcal) | 150 | Kcal | 150 | Kcal |
| Carbohydrate (g) | 15 | g | 15 | g |
| Fat(g) | 6 | g | 6 | g |
| Protein(g) | 9 | g | 9 | g |
| Vitamin A | 0 | μg RAE | 190 | μg RAE |
| Vitamin B_1_ | 0 | mg | 0.27 | mg |
| Vitamin B_2_ | 0 | mg | 0.3 | mg |
| Vitamin B_6_ | 0 | mg | 0.37 | mg |
| Vitamin C | 0 | mg | 30 | mg |
| Vitamin D | 0 | μg | 1.5 | μg |
| Vitamin E | 0 | mgTE | 20 | mgTE |
| Niacin | 0 | mg | 1.6 | mg |
| Folic acid | 0 | μg | 45 | μg |
| Calcium | 0 | mg | 160 | mg |
| Iron | 0 | mg | 3.2 | mg |
| Zinc | 0 | mg | 2.4 | mg |
| Magnesium | 0 | mg | 30 | mg |
| Phospholipid | 0 | mg | 200 | mg |
| Choline | 0 | mg | 480 | mg |
| Selenium | 0 | μg | 72 | μg |
| Vitamin B_12_ | 0 | μg | 0.75 | μg |
| Lutein | 0 | μg | 12 | μg |

Supplementary table 2. Comparison of ingredients between Placebo and Slikworm pillls

| **Placebo pills (2.5g)** | | | **Silkworm pills (2.5g)** | | |
| --- | --- | --- | --- | --- | --- |
| **Nutrient**  **Contents** | **Amount per daily dose** | **unit** | **Nutrient**  **Contents** | **Amount per daily dose** | **unit** |
| Energy | 9.5 | kcal | Energy | 10 | kcal |
| Carbohydrate | 2.20 | g | Carbohydrate | 1.20 | g |
| Fat | 0.05 | g | Fat | 0.16 | g |
| Protein | 0.07 | g | Protein | 0.94 | g |
| Moisture | 6.09 | % | Moisture | 5.22 | % |
| Ash | 1.12 | % | Ash | 2.8 | % |
